# INVESTIGATING THE RELATIONSHIP BETWEEN HABITUAL ACTIVITY AND COGNITIVE DOMAINS AMONG PEOPLE LIVING WITH DEMENTIA

**DOI:** 10.1093/geroni/igad104.3765

**Published:** 2023-12-21

**Authors:** Faheem Pottayil, Yanbin Dong, Haidong Zhu, Richard Sams, Lufei Young, Deborah Jehu

**Affiliations:** Medical College of Georgia, Marietta, Georgia, United States; Medical College of Georgia, Augusta, Georgia, United States; Medical College of Georgia, Augusta, Georgia, United States; Augusta University, Augusta, Georgia, United States; University of North Carolina at Charlotte, Charlotte, North Carolina, United States; Augusta University, Augusta, Georgia, United States

## Abstract

Habitual activity preserves cognitive function in people without dementia, but the relationship between habitual activity and cognitive domains among people living with dementia is unclear. The purpose of this study was to explore the association between habitual activity and cognition domains among people living with dementia. Participants living with dementia in residential care facilities (complete case analysis: n=24/42) completed a battery of cognitive tests (global cognition: Montreal Cognitive Assessment; executive function: Trail-Making Test, Digit Span Forward Test; perception and orientation: Benton Judgement of Line Orientation Test; language: Boston Naming Test; learning and memory: Rey Auditory Verbal Learning Test; complex attention: Digit Symbol Substitution Test). Participants wore an actigraphy monitor on their non-dominant wrist over seven days. We conducted a linear regression for total habitual activity (independent variable) with race (white/black), fall risk (Morse Fall Scale), and the number of comorbidities (Functional Comorbidities Index) as covariates, and cognitive tests as variables of interest. Participants were primarily male (75%), white (87.5%), and 50% had unspecified dementia (Alzheimer’s disease: 33%). Greater habitual activity was associated with poorer global cognition, better executive function, and better learning and memory (ps< 0.05). Habitual activity was not related to visuospatial perception, language, or complex attention. Habitual activity may preserve executive function and learning and memory among people living with dementia. Wandering is more common in later stages of dementia, which may explain greater habitual activity observed with lower global cognition. Regularly assessing habitual activity may be useful in screening and monitoring cognitive changes.

